# Physical activity, diet, and weight loss in patients recruited from primary care settings: An update on obesity management interventions

**DOI:** 10.1002/osp4.514

**Published:** 2021-05-04

**Authors:** Louise de Lannoy, Theresa Cowan, Angela Fernandez, Robert Ross

**Affiliations:** ^1^ School of Kinesiology and Health Studies Queen's University Kingston Ontario Canada; ^2^ School of Medicine Faculty of Health Sciences Queen's University Kingston Ontario Canada

**Keywords:** diet, physical activity, primary care, weight loss

## Abstract

**Background:**

Obesity and related comorbidities are the most common chronic conditions in North America where behavior modification including the adoption of physical activity (PA) and a healthful diet are primary treatment strategies. Patients are more likely to engage in behavior modification if encouraged by their physician; however, behavioral counseling in primary care rarely occurs due to lack of training and resources. A more effective method may be to refer patients from clinical settings to other health professionals.

**Objective:**

This systematic review examines the effectiveness of behavior‐based counseling for obesity management among participants referred from clinical settings.

**Methods:**

PubMed, CINAHL, and EMBASE were used to identify randomized clinical trials (2014–2020) for weight loss with the following inclusion criteria: trial duration ≥12 months, included a control or usual care group, recruited adults with overweight or obesity from primary care and/or treated in the primary care setting, and the intervention included counseling on PA and diet.

**Results:**

Seventeen studies, encompassing 21 different intervention groups with 6185 unique participants (56% female) met the inclusion criteria. All participants had overweight or obesity, with a body mass index between 28.2 and 41.0 kg/m^2^. In 11 (52%) of the intervention groups, significant weight loss in the intervention group was observed compared to usual care (mean weight loss: 4.9[2.1] kg vs. 1.0[0.9] kg). In 13 out of 18 interventions (72%) reporting weight loss at two time points, weight regain was observed by 12 months. Statistically significant weight loss was observed in one intervention (of two total) that was longer than 12 months.

**Conclusions:**

Sustained weight loss regardless of the behavior‐based, intervention strategy remains a challenge for most adults. Given the established benefits of routine PA and a healthful diet, prioritizing the adoption of healthy behaviors regardless of weight loss may be a more effective strategy for ensuring long‐term health benefit.

## 1 INTRODUCTION

Obesity and related comorbidities, such as diabetes and cardiovascular disease, are the most common chronic conditions in North America^1^ where behavior modification including the adoption of physical activity (PA) and a healthful diet are the primary treatment strategies.^1^ Patients are more likely to engage in behavior modification if encouraged by their physician^2^, ^3^ thus primary care clinics are an ideal setting for behavior‐based weight counseling.^4^ However, behavioral counseling in primary care rarely occurs, where only ∼20% of individuals with obesity receive advice on exercise and diet.^5^

The low rate of physician counseling has been attributable to several factors including a lack of training and resources^3^, ^4^, ^6^ as well as a general pessimism on the effectiveness of weight loss counseling.^5^, ^6^, ^7^ A more effective method may therefore be to refer patients from clinical settings to programs led by other trained health professionals (i.e., dietitians, lifestyle coaches, kinesiologists). In 2014, Wadden and colleagues published a systematic review^8^ that examined the effectiveness of behavior‐based counseling in which participants were recruited from clinical settings for weight management in 10 randomized clinical trials. The authors showed that the most effective weight loss interventions were those that combined diet, PA, and behavioral therapy, but also, that most studies showed weight regain after 12 months. In the 6 years since that publication, 17 new randomized behavior‐based weight loss studies have been published, highlighting the need to update this topic.

This review provides an update on the effectiveness of weight loss interventions in adults with overweight or obesity recruited from and/or treated in the primary care setting. Randomized controlled trials that combined diet and PA and were 12 months in duration or longer were included to examine patterns of weight loss over time.

## 2 MATERIAL AND METHODS

This systematic review was completed in adherence with the Preferred Reporting Items for Systematic Reviews and Meta‐Analyses (PRISMA).

### 2.1 Study selection

Articles were included in the current analysis if they met the following criteria: (1) the publication was written in English, (2) participants were adults with overweight or obesity, (3) the intervention protocol involved counseling on PA and diet and their role in weight loss, (4) the trial included either a control group, where no type of counseling on PA and diet occurred or there was a usual care comparator group, where participants only met with a health care provider for routine medical care visits and received behavioral advice according to routine practice, (5) weight was recorded at baseline and at minimum 12 months later, (6) the trial employed a randomized design, and (7) participants were recruited from and/or treated in the primary care setting.

### 2.2 Data sources

**FIGURE 1:**
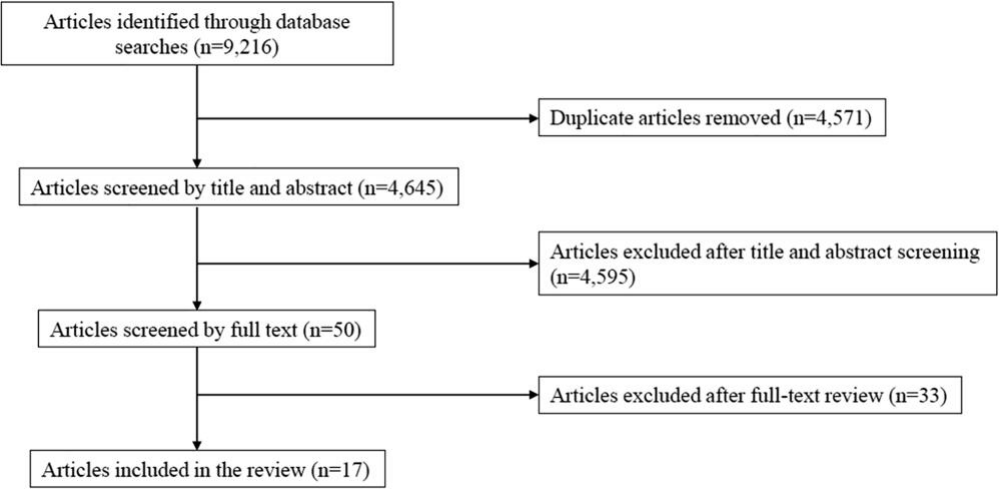
PRISMA flow diagram

Search strategies using PubMed, CINAHL, and EMBASE (2014–September 2020) were performed using the following terms: primary care, weight loss, counseling, lifestyle counseling, behavior modification, diet, exercise, and PA. These searches produced a total of 9216 titles and abstracts (Figure 1). Titles and abstracts were screened against the inclusion criteria. Potentially relevant articles were retrieved online or downloaded for further evaluation. Each abstract was reviewed by LDL, TC, and AF. Discrepancies were resolved by video conference call discussion between LDL, TC, and AF.

### 2.3 Data extraction

A standardized data extraction form was used to collect the following data: (1) trial characteristics: authors, date of publication, and trial design, (2) intervention characteristics: intervention type, duration, and intensity (contact frequency with intervention personnel), (3) participant characteristics: sample size, age, percentage female, weight, body mass index (BMI), socioeconomic status (SES), and ethnicity, and (4) outcome measures: body weight, waist circumference, PA, diet, cardiorespiratory fitness, systolic and diastolic blood pressure, glucose, insulin, and lipid measures, and quality of life scores.

### 2.4 Study Quality Assessment

The National Collaborating Centre for Methods and Tools' Quality Assessment Tool for Quantitative Studies^9^ was used to assess the quality of each study. This tool is used to rate studies as strong, moderate or weak across 8 categories (selection bias, study design, confounders, blinding, data collection methods, withdrawals and dropouts, intervention integrity, and analysis). The first six categories are used to calculate an overall rating: a strong rating was given to studies that had at least four strong ratings and no weak rating, a moderate rating was given to studies that had less than four strong ratings and 1 weak rating, a weak rating was given to studies that had two or more weak ratings. The Cochrane Collaboration's tool for assessing risk of bias^10^ was used to assess risk of bias of included studies. This tool is used to categorize risk of bias as either low, high or unclear risk using five categories: random sequence generation, allocation concealment, blinding of outcome assessment, incomplete outcome data, selective reporting.

### 2.5 Summary measures

Trial characteristics and results of individual interventions are presented in Table 1. Studies were identified as those in which statistically significant weight loss was achieved if the weight loss at follow‐up (i.e., at 12 or 24 months) was significantly different (*p* < 0.05) from the comparator group. Studies were identified as those in which statistically significant weight loss was not achieved if weight loss at follow‐up was not statistically different (*p* > 0.05) from the comparator group.

**TABLE 1:** Randomized controlled trials for weight loss

Paper	*N*	Study duration	Intervention groups	Female (%)	Age	BMI	Primary contact	Counselling style	Weight change baseline to 3–6 months (kg)	Weight change baseline to follow‐up (kg)
Ma et al. (2015)^11^	330	12 months	Usual care	117 (70.0)	47.7 (12.1)	37.6 (5.7)	PCP	‐	−1.1 (0.8)	−2.1 (0.8)
			DPP‐based diet + PA	116 (70.3)	47.5 (12.6)	37.4 (6.0)	Health educator	Combined one‐on‐one and group	−5.0 (0.8)	−4.0 (0.8)*
McRobbie et al. (2016)^12^	330	12 months	Usual care	75 (69.0)	45.1 (14.2)	35.7 (4.3)	Nurse practitioner	‐	−2.1 (4.3)	−2.3 (6.6)
			Diet + PA	161 (73.0)	46.6 (15.0)	35.0 (4.2)	Health educator	Combined one‐on‐one and group	−5.0 (5.4)	−4.2 (7.3)*
Moncrieft et al. (2016)^13^	111	12 months	Usual care	42 (77.8)	54.8 (6.3)	32.9 (5.5)	PCP	‐	−0.4	−1.4
			DPP‐based diet + PA	37 (64.9)	54.8 (8.3)	32.3 (3.7)	Trained therapist	Combined one‐on‐one and group	−3.3	−3.0*
Chee et al. (2017)^14^	230	12 months	Usual care	56 (48.7)	54 (8.0)	28.9 (6.3)	Dietitian, PCP	‐	−0.8 (0.5)	+0.5 (0.6)
			Diet + PA for diabetes management	50 (87.4)	55 (8.0)	29.4 (7.3)	Dietitian, PCP	One‐on‐one	−5.3 (1.2)	−3.3 (1.2)
			Diet + PA with motivational interviewing	39 (67.2)	55 (8.0)	30.7 (8.2)	Dietitian, PCP	One‐on‐one	−6.9 (1.3)	−5.8 (1.3)*
Johansen et al. (2017)^15^	98	12 months	Usual care	16 (47.0)	56.6 (8.1)	32.5 (4.5)	Nurse practitioner	‐	N/A	−2.0 (−4.0 to 0.1)
			Diet + PA	31 (48.0)	53.6 (9.1)	31.4 (3.9)	Dietician	Combined one‐on‐one and group	N/A	−6.1 (−7.5 to 4.7)*
Sellman et al. (2017)^16^	108	12 months	Usual care	46 (85.0)	42.4 (10.9)	40.8 (7.3)	PCP	‐	N/A	−0.7 (5.6)
			Diet + PA and obesity recovery treatment	45 (83.0)	45.1 (10.9)	41.0 (7.0)	Lifestyle coach	Combined one‐on‐one and group	N/A	−3.6 (6.5)*
Lean et al. (2018)^17^	298	12 months	Usual care	56 (38.0)	55.9 (7.3)	34.2 (4.3)	PCP	‐	N/A	−1.0 (3.7)
			Diet replacement + PA and food reintroduction	66 (44.0)	52.9 (7.6)	35.1 (4.5)	Nurse or dietitian	One‐on‐one	N/A	−10.0(8.0)*
Katzmarzyk et al. (2020)^18^	803	24 months	Usual care	280 (34.9)	50.1	37.2 (4.8)	PCP	‐	−0.8	−0.9
			DPP‐based	398 (50.0)	48.8	37.3 (4.6)	Health coach	One‐on‐one and group	−7.8	−5.4*
Bennett et al. (2018)^19^	351	12 months	Usual care	119 (68.0)	50.5	35.9 (3.7)	PCP	‐	0.3	−0.1
			Diet + PA	120 (68.2)	50.9	35.9 (4.1)	Dietitian, PCP	One‐on‐one over phone	−4.1	−4.0
Ma et al. (2019)^20^	409	12 months	Usual care	143 (69.8)	51.0	36.6 (5.8)	PCP	‐	0	0.2
			DPP‐based diet + PA	144 (70.6)	50.9	36.7 (6.9)	Health coach	One‐on‐one	−1.9	−2.6
Salas‐Salvado et al. (2019)^21^	626	12 months	Usual care	252 (84.3)	65.0	32.6 (3.6)	Dietitian	‐	−0.4	−0.7
			Diet + PA	282 (86.2)	66.0	32.3 (3.4)	Dietitian	One‐on‐one and group	−2.4	−3.2
Conroy et al. (2015)^22^	99	12 months	Usual care	49 (100)	54.0 (5.6)	33.4 (5.4)	PCP	‐	−1.1 (2.6)	−1.4 (3.8)
			DPP‐based diet + PA	49 (100)	53.8 (5.3)	36.1 (6.0)	PCP or interventionist	Group	−1.7 (4.0)	−1.4 (6.8)
Greaves et al. (2015)^23^	108	12 months	Usual care	14 (26.4)	63.7 (7.4)	32.3 (3.0)	Regular PCP contact	‐	−1.0 (3.6)	−2.0 (6.9)
			Diet + PA	19 (34.5)	66.6 (6.4)	33.0 (3.2)	Lifestyle coach	Group	−3.3 (3.5)	−4.3 (5.5)
Wennehorst et al. (2016)^24^	83	12 months	Usual care	27 (67.5)	53.3 (10.3)	32.1 (6.0)	PCP	‐	−0.5	−0.8
			Diet‐focused + PA	36 (83.7)	50.1 (6.1)	30.9 (6.4)	PCP or nutritionist	One‐on‐one	−4.5	−4.1
McInnes et al. (2017)^25^	83	12 months	Usual care	14 (50.0)	58.2 (11.1)	31.6 (4.4)	PCP	‐	−1.0	−1.6
			Diet + PA 8‐week program	14 (50.0)	55.1 (9.2)	34.7 (7.0)	Dietitian, kinesiologist	Combined one‐on‐one and group	−2.8	−0.1
			Diet + PA 16‐week program	15 (55.6)	57.9 (10.5)	33.3 (5.5)	Dietitian, kinesiologist	Combined one‐on‐one and group	−4.3	−1.9
Tapsell et al. (2017)^26^	377	12 months	Usual care	(74.0)	45 (37–51)	32 (29–35)	PCP	‐	−1.8	−4.0
			Diet + PA	(74.0)	45 (37–51)	32 (29–35)	Health coach, dietitian	One‐on‐one	−1.6	−5.4
			Diet + PA + 30 g walnuts/day	(74.0)	45 (37–51)	32 (29–35)	Health coach, dietitian	One‐on‐one	−3.1	−3.5
Ismail et al. (2019)^27^	1742	24 months	Usual care	82 (15.7)	70.0	28.4 (4.6)	PCP	‐	N/A	−0.2
			Diet + PA one‐on‐one	66 (12.6)	69.8	28.3 (4.3)	Healthy lifestyle facilitator	One‐on‐one	N/A	−0.8
			Diet + PA group	104 (14.9)	69.6	28.2 (4.1)	Healthy lifestyle facilitator	Group	N/A	−0.6

*Note:* Weight change indicated as mean (±SD) or (95% confidence interval) where available.

Abbreviations: DPP, diabetes prevention program; PA, physical activity; PCP, primary care physician.

*Indicates statistically significant difference (*p* < 0.05) compared to the usual care group.

## 3 RESULTS

### 3.1 Study selection

There were a total of 9216 titles and abstracts screened. Seventeen studies consisting of 21 intervention groups met the inclusion criteria (Figure 1; Table 1).

### 3.2 Study quality and risk of bias assessment

Study quality was evaluated using the Quality Assessment tool for Quantitative Studies.^9^ All studies were rated as high quality. Risk of bias was assessed using the Cochrane Collaboration's tool for assessing risk of bias.^10^ All studies were identified as having low risk of bias across all five bias assessment categories.

### 3.3 Study characteristics—Overview

The 17 included studies encompassed a total of 6185 participants where the duration of the intervention ranged from 12 to 24 months (Table 1). Female participants accounted for 56% of all participants. The average age of participants ranged from 42.4 ± 10.9 years^16^ to 70.0 ± 4.1 years.^27^ All participants were categorized as having overweight or obesity with an average BMI > 28 kg/m^2^.

In two studies (12.0% of the total study population [*n* = 724] reviewed here)^15^, ^21^ the authors did not report the ethnicity of participants though the studies were performed in Spain^21^ and the Netherlands.^15^ In the remaining studies that included information on ethnicity, 75% of participants were identified as White, 20% were Black, 15% were identified as Asian or other, and 6% were Hispanic/Latino.

The SES of participants varied across studies. Most studies included participants from a range of incomes and educational levels. There were three studies^11^, ^20^, ^23^ in which the majority of participants had a high SES and four studies^13^, ^18^, ^19^, ^21^ that recruited participants with a low SES. For example, more than 70% of the participants in the study by Ma et al.^20^ earned >$75,000/year, whereas in the study by Moncrieft et al.^13^ the average income of participants was $14,000.

### 3.4 Study characteristics—Primary contact

All studies involved behavioral‐based counseling designed to encourage participants to decrease weight and improve cardiometabolic outcomes. In four studies^14^, ^19^, ^22^, ^24^ the primary care physician (PCP) together with another health care provider (dietician, nutritionist or interventionist) were the primary contacts for the delivery of the intervention (Table 1). In the studies that did not have the PCP as the primary contact, the intervention was led by either a lifestyle coach,^16^, ^18^, ^20^, ^23^, ^26^ health educator,^11^, ^12^, ^27^ nurse,^17^ dietician,^15^, ^17^, ^21^, ^25^, ^26^ kinesiologist,^25^ or therapist^13^ trained or experienced in delivering the counseling materials.

### 3.5 Study characteristics—Group versus one‐on‐one counseling

Interventions included one‐on‐one counseling,^14^, ^17^, ^19^, ^20^, ^24^, ^26^, ^27^ group sessions,^22^, ^23^, ^27^ or a combination of one‐on‐one and group sessions^11^, ^12^, ^13^, ^15^, ^16^, ^18^, ^21^, ^25^ **(**Table 1 **)**. One‐on‐one based interventions provided participants with tailored counseling on diet and PA and were often adjusted to fit the needs of the individual by modifying personal goals throughout the intervention. Most one‐on‐one and group sessions were performed in‐person, however, seven of the one‐on‐one interventions were delivered in part by telephone,^11^, ^12^, ^19^, ^20^, ^21^, ^25^, ^26^ and two interventions included email and text messages.^12^, ^16^

### 3.6 Study characteristics—Diet versus exercise

All interventions included caloric restriction and/or recommendations to improve dietary quality and increase PA for weight loss. Five^11^, ^13^, ^18^, ^20^, ^22^ interventions were adapted from the diabetes prevention program^28^ and as such included the following general goals for participants: achieve a minimum of 150 min of moderate intensity PA per week, reduce dietary fat intake to less than 25% of calories, and attain 5%–10% body weight loss. In four interventions,^17^, ^21^, ^24^, ^26^ more emphasis was placed on dietary changes over increasing PA. Among these four interventions, three^21^, ^24^, ^26^ encouraged participants to make healthier food choices such as eating more plant‐based foods, whereas Lean et al.^17^ prescribed a very low energy diet with gradual increase in intake over time.

### 3.7 Study results—Weight loss

In 11 of the 21 interventions (52%),^11^, ^12^, ^13^, ^14^, ^15^, ^16^, ^17^, ^18^, ^19^, ^20^, ^21^ statistically significant weight loss was observed in the intervention group compared to usual care **(**Table 1 **).** In three of these interventions, the weight loss achieved was greater than 5%.^14^, ^17^, ^18^ In seven interventions weight change was reported for two time points (6 and 12 months)^11^, ^12^, ^13^, ^14^, ^19^, ^20^, ^21^; in five (71%) of these interventions weight loss at 6 months was greater than at 12 months **(**mean weight lost from baseline at 6 and 12 months: 4.1 and 3.8 kg, respectively). Statistically significant weight loss was achieved in one out of two interventions reviewed here that were longer than 12 months in duration (Table 1).

In 10 of the 21 intervention groups (48%), statistically significant weight loss was not observed at the end of the intervention compared to usual care^14^, ^22^, ^23^, ^24^, ^25^, ^26^, ^27^ (Table 1 **).** All 10 intervention groups reported on weight change at two time points. In five (50%) of these interventions, weight loss at 3–6 months was greater than at 12 **(**mean weight lost from baseline at 3, 6 and 12 months: 2.3, 4.2 and 2.5 kg, respectively). One study^27^ was longer than 12 months in duration; weight regain was also observed, where weight loss at 12 months was greater than at 24 months.

### 3.8 Study results—Participant characteristics

The interaction between sex and weight loss was considered in two studies^11^, ^13^; sex did not modify weight loss in either study. Most studies in which there was significant weight loss included participants from a wide range of incomes and educational levels, though in two of these studies, participants had a high SES^11^, ^20^ and three others recruited participants with low SES.^13^, ^18^, ^19^ There were no observable differences between participants in terms of age, baseline BMI, or ethnicity in studies in which significant weight loss was achieved compared to studies in which significant weight loss was not achieved.

### 3.9 Study results—Primary contact

In almost all trials in which statistically significant weight loss was observed^11^, ^12^, ^13^, ^14^, ^15^, ^16^, ^17^, ^18^, ^19^, ^20^, ^21^ a health care provider other than the PCP was the primary contact for the delivery of the intervention (Table 1), with the exception of the trial by Chee et al.^14^ in which the PCP together with a dietician were the primary contacts. In trials in which non‐significant weight loss was reported,^14^, ^22^, ^23^, ^24^, ^25^, ^26^, ^27^ either a PCP or another health care provider were the primary contacts (Table 1).

### 3.10 Study results—Contact with interventionists over time

On average, participants met (either by phone or in‐person) with an interventionist 23 (SD: 44) times over the course of the intervention: 16 (SD: ±29) times in the first 6 months, and 7(15) times in the following 6 months. In trials in which significant weight loss was reported,^11^, ^12^, ^13^, ^14^, ^15^, ^16^, ^17^, ^18^, ^19^, ^20^, ^21^ participants met with an interventionist on average 22(38) times in the first 6 months, and 11(20) times in the last 6 months. In trials in which non‐significant weight loss was reported,^14^, ^22^, ^23^, ^24^, ^25^, ^26^, ^27^ participants met with an interventionist on average 10(3) times in the first 6 months, and 1(2) time in the last 6 months.

### 3.11 Study results—Intervention design

All interventions in which there was statistically significant weight loss employed one‐on‐one^14^, ^17^, ^19^, ^20^ or a combination of one‐on‐one and group^11^, ^12^, ^13^, ^15^, ^16^, ^18^, ^21^ counseling techniques **(**Table 1). In one intervention participants were prescribed a very low energy diet,^17^ whereas the majority of interventions in which significant weight loss was observed prescribed a healthful diet and/or moderate calorie restriction (ex. 500–1000 kcal/day) caloric restriction based on body weight, to consume no less than 1200 kcal/day together with PA at^11^, ^13^, ^14^, ^15^, ^16^, ^19^, ^20^ or above^17^, ^18^, ^21^ the consensus recommendation (150 min/wk of moderate‐to‐vigorous intensity PA).

### 3.12 Study results—Health benefits beyond weight loss

Of the 21 intervention groups reviewed, there were 17 interventions^11^, ^13^, ^14^, ^15^, ^16^, ^17^, ^18^, ^19^, ^20^, ^21^, ^23^, ^25^, ^26^, ^27^ in which improvement in secondary measures was observed **(**Table 2 **)**. For example, in eight interventions there was an improvement in glycemic control,^13^, ^14^, ^15^, ^17^, ^21^, ^25^ in five interventions there was an improvement in blood pressure,^14^, ^19^, ^25^, ^27^ in seven interventions there was an improvement in quality of life,^13^, ^16^, ^17^, ^18^, ^20^, ^26^ and in four interventions there was an improvement in leisure‐time PA.^11^, ^21^, ^26^ Improvement in these measures were observed in interventions in which both significant and non‐significant weight loss was reported. The study by Chee et al.^14^ was the only study that explored interactions between weight loss and cardiometabolic improvement; the authors showed that the greater the weight loss, the greater the improvement in HbA1c levels.

**TABLE 2:** Statistically significant improvement in secondary outcomes in randomized controlled trials for weight loss

Paper	Secondary outcomes						
	Glycemic Control and/or reduced glucose‐lowering medication usage	Systolic blood pressure	Diastolic blood pressure	Waist circumference	Regular physical activity	Diet quality	Quality of life
Significant weight loss reported							
Ma et al. (2015)^11^				✔	✔		
McRobbie et al. (2016)^12^							
Moncrieft et al. (2016)^13^	✓						✓
Chee et al. (2017)^14^; Intervention 1	✓	✓		✓			
Chee et al. (2017)^14^; Intervention 2	✓	✓	✓				
Johansen et al. (2017)^15^	✓						
Sellman et al. (2017)^16^							✓
Lean et al. (2018)^17^	✓						✓
Bennett et al. (2018)^19^		✓	✓	✓			
Ma et al. (2019)^20^							✓
Salas‐Salvado et al. (2019)^21^	✓			✓	✓		
Katzmarzyk et al. (2020)^18^				✓			✓
No significant weight loss reported							
Conroy et al. (2015)^22^							
Greaves et al. (2015)^23^				✓		✓	
Wennehorst et al. (2016)^24^							
McInnes et al. (2017)^25^ Intervention 1	✓						
McInnes et al. (2017)^25^ Intervention 2	✓		✓				
Tapsell et al. (2017)^26^ Intervention 1					✓	✓	✓
Tapsell et al. (2017)^26^ Intervention 2					✓	✓	✓
Ismail et al. (2019)^27^, Intervention 1			✓				
Ismail et al. (2019)^27^, Intervention 2							

## 4 DISCUSSION

The primary finding from this review is that, although statistically significant weight loss was reported in over half of the behavioral‐based interventions, weight regain was observed in most regardless of whether significant weight loss was achieved. This suggests that sustained behavioral‐based weight loss in primary care settings continues to be a challenge suggesting the need for a revised strategy.

The 2013 American Heart Association guidelines for overweight and obesity^29^ state that combining diet, PA, and behavioral modification techniques together is an effective approach for clinically meaningful weight loss (5%–10% reduction in baseline body weight). Similar observations were made in the 2018 Evidence Report by the US Preventive Services Task Force on Behavioral and Pharmacotherapy Weight Loss interventions.^30^ However, similar to previous findings,^8^, ^31^, ^32^ the majority of the behavioral‐based intervention groups included in this review did not achieve the 5%–10% weight loss threshold; on average, intervention groups lost 3%–4% of baseline body weight. Moreover, most trials were only 12 months in duration and exhibited weight regain by the end of the trial. It is possible that had the follow‐up been longer, few studies would have exhibited significant weight loss. The challenge of obesity management remains sustained weight loss in today's obesogenic environment.

Among those trials in which significant weight loss was observed at follow‐up, the findings reveal that most provided high frequency, one‐on‐one contact with interventionists. The importance of these study design components have been echoed in previous reviews^3^, ^8^, ^33^ including the 2013 National Institutes of Health Review on Management of Overweight and Obesity^34^ and an updated review on behavior modifications by Wadden et al.^35^ This review confirms and extends these observations by highlighting that not only was frequency of contact important, but that in most studies, the decrease in contact frequency in the second half of the intervention coincided with weight regain. This suggests that maintaining healthful behaviors associated with weight loss is difficult without continued, intensive support. Frequent contact with patients may not be achievable for many PCPs and thus it is encouraging that the majority of studies reporting significant weight loss employed a health care provider other than a PCP to deliver the intervention. While there is some evidence that brief counseling from PCPs can be effective in promoting weight loss,^36^ lack of training and resources continues to be a challenge for many PCPs.^3^, ^4^, ^6^ Thus it is encouraging that effective weight management involving frequent one‐on‐one patient contact may be achieved without overburdening the PCP.

An alternative to primary care‐based weight loss may be referral to commercial weight loss clinics to maintain contact with patients long‐term. Recent research^34^, ^37^ suggests that referrals from PCPs to commercial weight loss clinics may be a practical alternative for obesity management especially if clinicians do not have the time or resources to implement an intervention in their own practice. However, a major limitation of commercial programs is the cost; these programs are costly and therefore inaccessible for populations that are often in the greatest need.^38^ Other strategies for helping individuals manage weight and weight loss include the use of technology (email, fitness trackers); however, the benefit of these devices for long‐term sustained weight loss remains uncertain.^39^ Fitness trackers may also be cost prohibitive for some. Moreover, recent evidence from the National Institutes of Health and others has suggested that fully automated weight loss programming is less effective than in‐person delivery^34^, ^40^ or a combined approach.^41^ Nonetheless, given the general accessibility of these technologies and the opportunities they provide in tailoring weight management programming to the individual, these resources may play a central role in future obesity counseling.

It is noteworthy that improvement in cardiometabolic variables was reported in over 80% of studies reviewed. This is encouraging as substantial evidence exists on the long‐term benefit of consuming a healthful diet and engaging in regular PA regardless of weight loss.^30^, ^42^, ^43^, ^44^, ^45^ Refocusing efforts away from weight loss and towards engaging in healthy behaviors as a measure of treatment efficacy is an important public health message. This does not imply that behavior‐based weight loss should not be recognized as a measure of treatment success. Rather, that the health benefit of behavior change can manifest in several ways and as such provides opportunity for physicians to assess and monitor successful obesity management using measures other than the weigh scale.

Strengths of this review include the use of PRISMA in conducting the search. In this review there were only two studies that were longer than 12 months in duration. The study by Katzmarzyk et al.^18^ is of particular importance given that significant weight loss was observed at 24 months and participants were from an underserved, low‐income population in the United States that typically face major barriers to effective obesity treatment. However, intervention participants received pre‐packaged foods and meal replacement products, which is likely cost‐prohibitive for this population and in most primary care settings. Given the dearth of knowledge on how to effectively support marginalized, low‐income communities, future research and policy efforts are required, especially to address bias and stigma that may otherwise perpetuate weight‐related challenges.^46^ Additional long‐term studies are needed to identify the most important and economically feasible contributors to successful long‐term obesity management.

## 5 CONCLUSION

The findings here reinforce the earlier findings of Wadden et al. and suggest that most adults are not able to sustain the major changes in behavior that are required to maintain weight loss long term. Given the established benefits of consuming a healthful diet combined with the adoption of PA, perhaps the time has come for practitioners to prioritize the adoption of healthy behaviors regardless of weight loss.

## CONFLICT OF INTEREST

The authors have no competing interests to declare.

## AUTHOR CONTRIBUTIONS

Louise de Lannoy, Theresa Cowan, and Angela Fernandez conducted the literature search and extracted, analyzed, and interpreted the data. All authors were involved in developing the study design, writing the paper, and had final approval of the submitted and published versions.
